# The ABCD of portal vein thrombosis: a systematic approach

**DOI:** 10.1590/0100-3984.2019.0109

**Published:** 2020

**Authors:** Alexandre Makoto Minoda, Raissa Brito Fernandes Cadete, Sara Reis Teixeira, Valdair Francisco Muglia, Jorge Elias Junior, Andréa Farias de Melo-Leite

**Affiliations:** 1 Instituto de Medicina Integral Professor Fernando Figueira (IMIP), Recife, PE, Brazil.; 2 Faculdade de Medicina de Ribeirão Preto da Universidade de São Paulo (FMRP-USP), Ribeirão Preto, SP, Brazil.; 3 Universidade Federal de Pernambuco (UFPE), Recife, PE, Brazil.

**Keywords:** Portal vein, Venous thrombosis, Hypertension, portal, Collateral circulation, Magnetic resonance imaging, Veia porta, Trombose venosa, Hipertensão portal, Circulação colateral, Ressonância magnética

## Abstract

Portal vein thrombosis refers to complete or partial obstruction of the portal venous system, in the intrahepatic or extrahepatic venous tract or even in the splenic or superior mesenteric veins. This common and potentially fatal condition can develop in various clinical contexts, especially those of liver cirrhosis, hepatocellular carcinoma, and other solid tumors. Certain characteristics, such as the time since the onset of the thrombus (acute or chronic), its biology (hematic or tumoral), the presence of collateral vessels, and the magnetic resonance imaging aspects, are important components of a thorough, careful analysis, as well as informing decisions regarding the appropriate therapeutic strategy. Here, we present a brief review of the anatomy of the portal venous system and a systematic approach to analyzing the condition, using a mnemonic (ABCD, for age, biology, collaterals, and diffusion). We discuss the various imaging methods and illustrate our discussion with images selected from the case files archived at our facility.

## Abstract (pt)

Trombose da veia porta refere-se à obstrução completa ou parcial do sistema venoso portal, localizada nos tratos venosos intra-hepáticos ou extra-hepáticos e até mesmo nas veias esplênica ou mesentérica superior. Vários contextos clínicos podem ser responsáveis pelo desenvolvimento desta condição frequente e potencialmente fatal, especialmente a cirrose hepática, o carcinoma hepatocelular e outros tumores sólidos. Algumas características como o tempo de aparecimento do trombo (agudo ou crônico), sua biologia (hemático ou tumoral), a presença de vasos colaterais e o seu comportamento na ressonância magnética são importantes para uma análise completa e criteriosa, assim como para o gerenciamento adequado da estratégia terapêutica. No presente artigo apresentamos breve revisão da anatomia do trato venoso portal, seguida de uma abordagem sistemática usando um mnemônico (ABCD) para análise da trombose da veia porta por diferentes métodos de imagem, utilizando imagens de casos selecionados do arquivo de ensino do nosso serviço.

## INTRODUCTION

Portal vein thrombosis is the most common cause of prehepatic portal hypertension, classically defined as partial or complete obstruction of the portal vein lumen. Anatomically, it can occur in the intrahepatic or extrahepatic portal venous tract and can involve the superior mesenteric vein, the splenic vein, or both^(1,2)^. Factors that compose the Virchow triad (hypercoagulability, endothelial dysfunction, and stasis), especially liver cirrhosis and neoplastic conditions, primarily hepatocellular carcinoma (HCC), can predispose to portal vein thrombosis^(1,3)^. The clinical presentation is quite variable, and patients can even be asymptomatic, which limits its diagnosis and classification when only clinical findings are considered^(3)^.

Ultrasound, computed tomography (CT), and magnetic resonance imaging (MRI) are the imaging methods used in order to facilitate the diagnosis and classification of portal vein thrombosis, with rates of sensitivity and specificity ranging from 80% to 100%^(4,5)^. When portal vein thrombosis is suspected, ultrasound is the first-line method, with an estimated accuracy of 88-98%, CT or MRI being used in patients with an inadequate acoustic window or when there is a need for better assessment of the extent of the thrombus and the portosystemic collaterals, as well as for the detection of abdominal neoplasms^(2,5,6)^.

In this article, we present a systematic approach using a mnemonic (ABCD, for age, biology, collaterals, and diffusion). The objective is to enable a complete analysis of portal venous thrombosis, regardless of the imaging employed.

## ANATOMY OF THE PORTAL VENOUS SYSTEM

**Figure 1:**
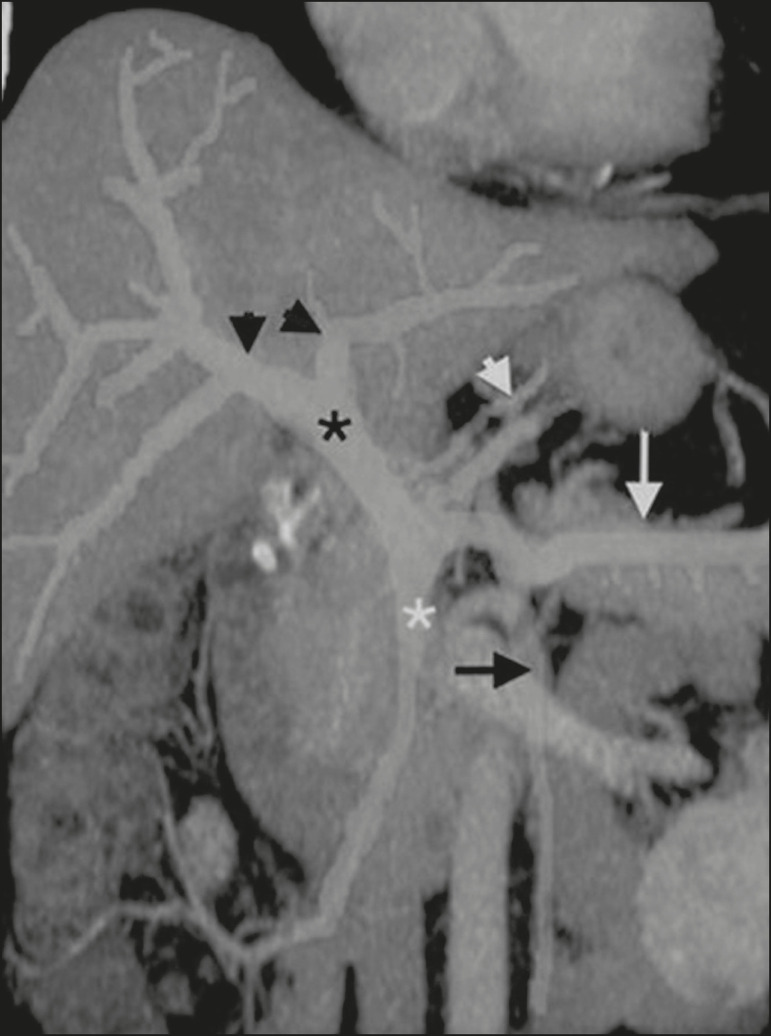
Coronal CT reconstruction showing the portal venous system. The main portal vein (black asterisk) is formed by the union of the splenic vein (white arrow) and the superior mesenteric vein (white asterisk). The right and left portal branches divide within the hepatic hilum (black arrowheads). Other tributaries include the inferior mesenteric vein (black arrow) and the gastric veins (white arrowhead).

**Figure 2:**
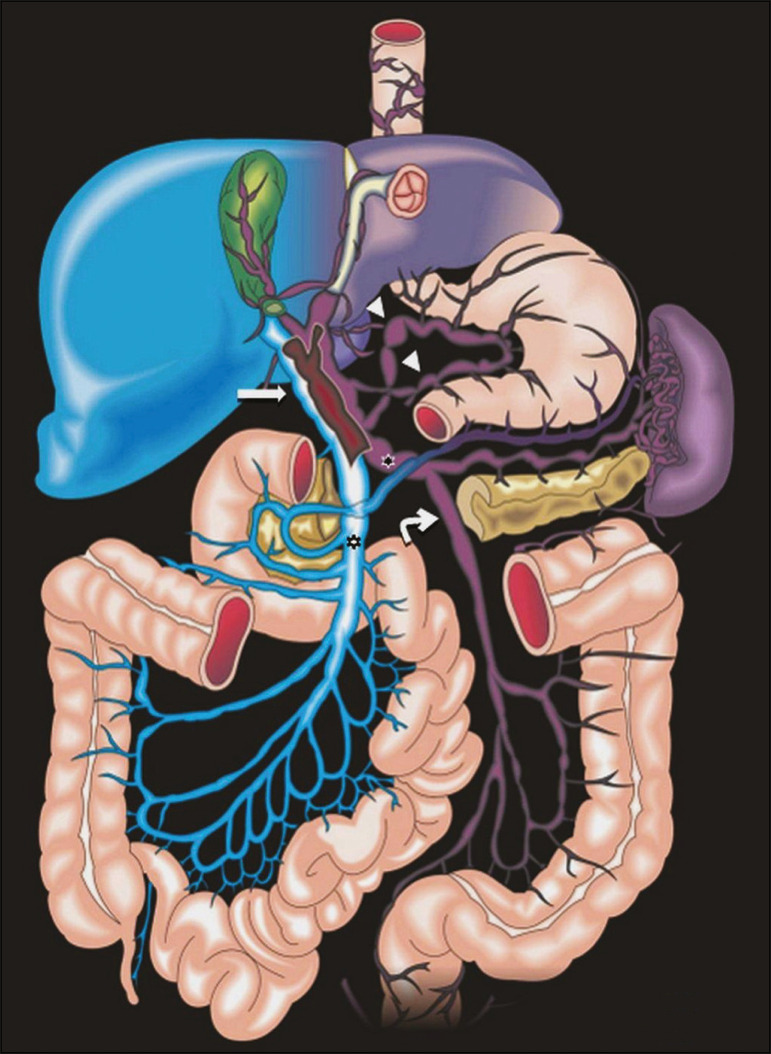
Schematic drawing showing the anatomy of the portal venous system. The portal vein (straight arrow), formed by the union of the splenic vein (black asterisk) and superior mesenteric vein (white asterisk), directly receives the right and left gastric veins (arrowheads). The inferior mesenteric vein (curved arrow) drains directly into the splenic vein. (Adapted from Netter^(10)^).

The portal vein forms posteriorly to the neck of the pancreas, at the junction of the splenic and superior mesenteric veins^(7,8)^. In the hepatic hilum, it divides into right and left branches, which insert into the liver, projecting a portal branch into the center of each hepatic segment (Figure 1). The splenic vein originates from the junction of its tributaries in the splenic hilum and receives the short gastric, left gastroepiploic, inferior mesenteric, and posterior pancreatic veins. The superior mesenteric vein is formed by tributaries that drain the colonic segments on the right, the small intestine, and the head of the pancreas. The left gastric vein connects directly to the portal vein at its origin. Other tributaries, such as the right gastric, cystic, accessory pancreatic, and superior pancreaticoduodenal veins, are also received by the portal vein^(7-9)^, as depicted in Figure 2.

## SYSTEMATIC RADIOLOGICAL APPROACH TO PORTAL VEIN THROMBOSIS

### A (age)

Portal vein thrombosis is classified as acute or chronic depending on the time since its onset. However, it is difficult to define the time of onset on the basis of clinical parameters. In addition to fact that it can be asymptomatic, the duration of symptoms has been shown to be an unreliable indicator of the time of onset of the condition^(5)^. Therefore, the use of imaging methods is a feasible and indispensable approach to making the differentiation between acute and chronic cases of portal vein thrombosis.

**Figure 3:**
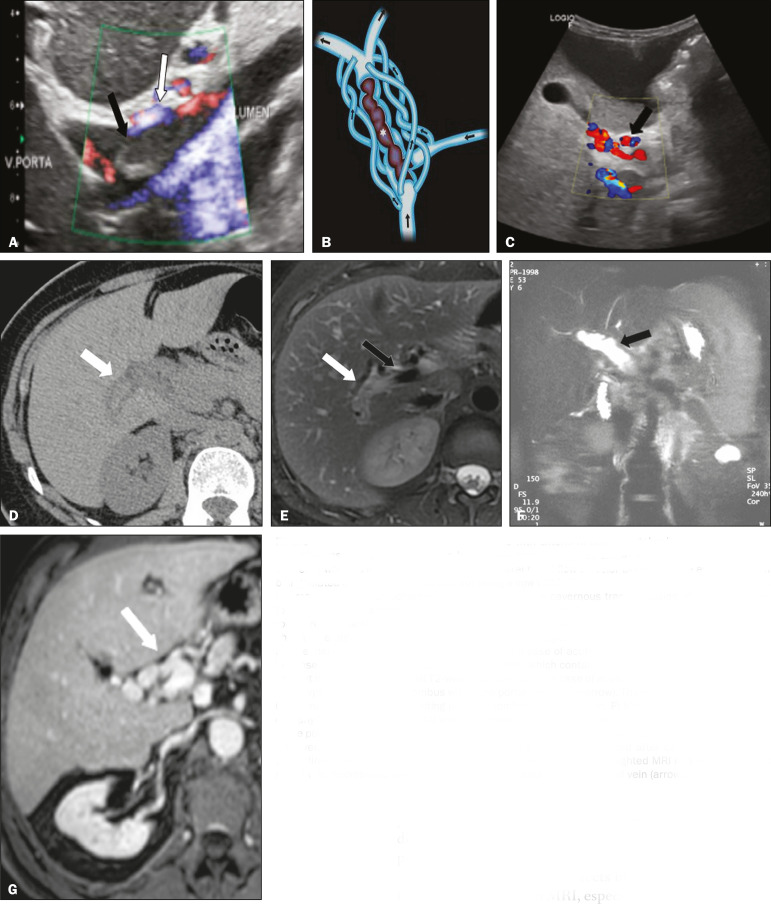
**A:** Patient in a hypercoagulable state with extensive acute portal vein thrombosis. Longitudinal ultrasound image showing a hypoechoic thrombus (black arrow) in the main portal vein, the caliber of which is increased, with slight peripheral blood flow on color Doppler (white arrow), the flow being related to partial occlusion and not being a flow within the thrombus (which differentiates it from a tumor thrombus). **B:** Schematic drawing showing the cavernous transformation of the portal vein. Extensive thrombus (asterisk) in the main portal vein, with the formation of multiple collaterals (arrows) around the obstruction. **C:** Color Doppler ultrasound in a case of chronic portal vein thrombosis, showing serpiginous vessels (arrow) in the periportal region, indicative of cavernous transformation. **D:** Unenhanced axial CT scan of the abdomen in a case of acute portal vein thrombosis, showing na increase in the caliber of the portal vein (> 13 mm), which contains hyperdense material, best seen in its right branch (arrow). **E:** Axial T2-weighted MRI scan in a case of acute portal vein thrombosis, showing a hyperintense acute thrombus within the portal vein (white arrow). There is flow artifact (flow void) in the main portal vein, suggesting partial thrombosis (black arrow). **F:** Magnetic resonance cholangiography in a case of acute portal vein thrombosis. Note the markedly hyperintense signal, suggesting acute portal vein thrombosis. Note also that, at first glance, it can mimic the common bile duct (arrow). However, the diagnosis of acute portal vein thrombosis was confirmed after detailed analysis and three-dimensional reconstructions. **G:** Contrast-enhanced axial T1-weighted MRI in a case of chronic portal vein thrombosis, showing cavernous transformation of the portal vein (arrow).

On ultrasound, an acute thrombus usually appears as heterogeneous material in the vessel lumen, accompanied by an increase in the caliber of the portal vein (to > 13 mm). In some cases, the thrombus is hypoechoic or isoechoic, and, on color Doppler, there is an absence of blood flow in part or all of the lumen^(9,11)^, as shown in Figure 3A. As the thrombus ages and becomes chronic, its content becomes more echogenic, with calcifications or cavernous transformation, which is defined as the formation of collateral vessels that bypass the obstruction, exhibiting multiple serpiginous structures with flow on color Doppler, in the periportal region^(9,11)^, as can be seen in Figures 3B and 3C. Despite being characteristic of the chronic phase, the appearance of a thrombus at 6-20 days after the acute event has been reported^(11)^. In the absence of cirrhosis or other concomitant causes of portal hypertension, signs of severe portal hypertension, such as ascites, splenomegaly, and portosystemic collaterals, also suggest chronicity.

On CT, the acute form of portal vein thrombosis presents as increased portal vein attenuation in the pre-contrast phase, a lack of enhancement after administration of intravenous contrast, and increased portal vein caliber^(4)^, as illustrated in Figure 3D. In the chronic form, the thrombosed vessel can be hypodense, containing linear calcifications, or obliterated, with cavernous transformation^(4,11)^. In candidates for liver transplantation, calcifications in the portal vein should be actively investigated, because they indicate a more fragile vessel and can complicate the surgical procedure^(4,5)^.

Total or partial filling defects in the portal venous system can be detected on MRI, especially in the portal phase. On T1-weighted images, acute thrombi can be isointense to the muscle tissue or hyperintense when newly formed, whereas they continue to show high signal intensity on T2-weighted images (Figure 3E). Chronic thrombi tend to be hypointense or appear as a flow void, although a slow flow artifact can change the signal to isointense or hyperintense^(6)^, as shown in Figures 3F and 3G.

### B (biology)

Thrombi can be differentiated, in terms of biology or nature, as having a vascular or tumor nature. Vascular thrombi are usually secondary to slow flow in patients with cirrhosis, whereas tumor thrombi are related to invasion of a portal branch by a neoplasm. Despite the disparate pathophysiology, the two types can coexist, especially in patients with HCC^(5)^. Tumor thrombosis is a common, well-reported complication of HCC and is considered one of the contraindications to liver transplantation, dramatically altering the treatment options and prognosis^(2,4,5)^.

**Figure 4:**
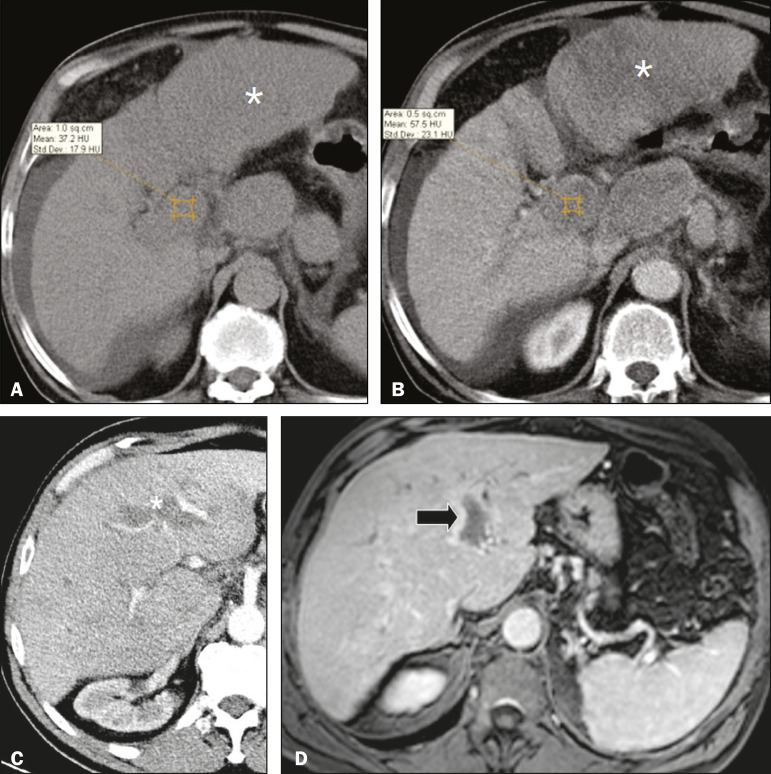
**A,B:** CT scan showing a tumor thrombus secondary to HCC (asterisk). Axial sections acquired before and after contrast administration (A and B, respectively), showing 20 UH of enhancement in the thrombus (i.e., 37 UH in the pre-contrast phase and 57 UH in the post-contrast phase). In addition, there is expansion of the vessel, also related to the tumor component. **C:** Axial CT scan of a patient with a vascular thrombus, showing no enhancement of the thrombus in the portal phase (asterisk). **D:** Contrast-enhanced T1-weighted MRI scan of a patient with a vascular thrombus in the left portal vein branch (black arrow), showing no enhancement.

On color Doppler, a tumor thrombus shows blood flow in its interior, with 100% specificity when the flow found is arterial (neovascularization), a finding that is absent in vascular thrombi^(11)^. Neovascularization on CT is also highly specific and has been widely described as indicative of a tumor thrombus, which typically shows enhancement greater than 20 HU after contrast administration^(12-14)^, as depicted in Figures 4A and 4B. Vascular thrombi show variable density, depending on their age, and do not show enhancement after contrast^(7)^, as can be seen in Figures 4C and 4D.

### C (collaterals)

The absence of portosystemic collaterals and splenomegaly usually indicates acute thrombosis, although the presence of those signs does not automatically indicate chronic thrombosis, because they can be attributable to intrahepatic portal hypertension secondary to cirrhosis^(5)^.

**Figure 5:**
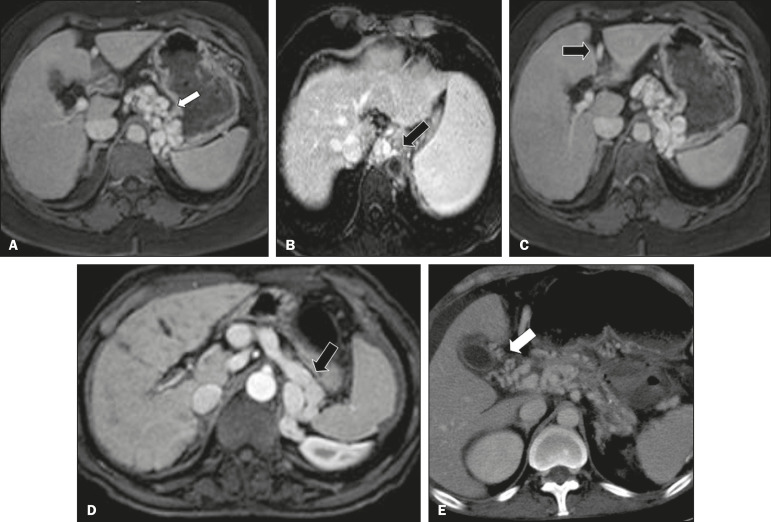
**A:** Gastroesophageal varices. Axial contrast-enhanced CT scan showing dilated vessels at the lesser curvature and at the gastroesophageal junction (arrow). **B:** Contrast-enhanced MRI showing paraesophageal varices, presenting as dilated vessels around the distal esophageal portion (arrow). **C:** Contrastenhanced axial T1-weighted MRI scan showing recanalization of the paraumbilical veins, the reopening of the veins occurring in the round/falciform ligament (arrow). **D:** Contrast-enhanced MRI of a splenorenal shunt. There is communication between the splenic vein and the left renal vein through serpiginous, dilated vessels (arrow). **E:** Axial contrast-enhanced CT scan showing dilated and tortuous vessels in the gallbladder wall (arrow).

The left gastric vein is responsible for the most common portosystemic shunt (Figure 5A), and a left gastric vein caliber > 6 mm is indicative of portal hypertension. Dilated vessels in the esophageal wall are known as esophageal varices, whereas those located adjacent to the esophagus are known as paraesophageal varices^(8)^, as illustrated in Figure 5B.

In patients with portal hypertension, a recanalized umbilical vein can also be observed (Figure 5C), as can a splenorenal shunt^(9)^, as shown in Figure 5D, and gallbladder wall varices, which are commonly seen in patients with cavernous transformation^(8)^, as depicted in Figure 5E. Other, less common shunts are deviation of the superior mesenteric vein to the right renal vein, mesenteric varices, and collaterals to the diaphragm.

### D (diffusion)

Diffusion-weighted imaging (DWI) can facilitate the qualitative and quantitative evaluation of portal vein thrombosis. The role of DWI in the differentiation between vascular and tumor thrombi was emphasized by Catalano et al.^(6)^, who demonstrated that tumor thrombi have signal intensity and apparent diffusion coefficient (ADC) values similar to those of the primary tumor, the signal being hyperintense on DWI, which shows restriction, and hypointense on the ADC map^(5,6)^, as depicted in Figures 6A and 6B. Vascular thrombi do not typically present restricted diffusion, except in cases of acute hemorrhage, in which there is a “shine-through” effect with different hemoglobin degradation products, limiting and confounding the accurate analysis of the sequence. Therefore, DWI is an additional tool that, together with the determination of other characteristics, such as the caliber of the portal vein, tumor expansion, neovascularization, the signal in the other (T1- and T2-weighted) sequences, and the signs of portal hypertension, helps differentiate between vascular and tumor thrombi^(5,13,14)^.

**Figure 6:** **A:** DWI of a patient with chronic liver disease and diffuse HCC, showing restricted diffusion in the right hepatic lobe (white arrow) and in the left portal vein branch (black arrow). **B:** DWI of another patient with chronic liver disease and diffuse HCC, in which the ADC measured in the thrombus was 1.0 × 10^−3^ mm^2^/s, a value similar to that found for the tumor.


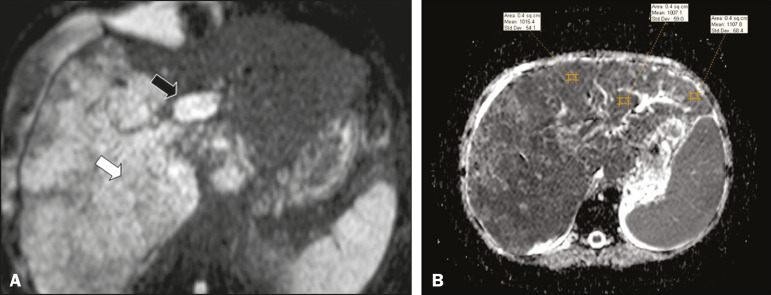


## CONCLUSION

Because of its morbidity and the limitations of the clinical evaluation, portal vein thrombosis demands careful analysis by imaging methods. Important information should be extracted by the various imaging methods. By adopting the systematic ABCD approach, the data can be interpreted in a more careful and comprehensive manner.
